# *In vivo* bistatic dual-aperture ultrasound imaging and elastography of the abdominal aorta

**DOI:** 10.3389/fphys.2024.1320456

**Published:** 2024-03-28

**Authors:** Vera H. J. van Hal, Hein de Hoop, Marc R. H. M. van Sambeek, Hans-Martin Schwab, Richard G. P. Lopata

**Affiliations:** ^1^ Photoacoustics and Ultrasound Laboratory Eindhoven (PULS/e), Department of Biomedical Engineering, Eindhoven University of Technology, Eindhoven, Netherlands; ^2^ Department of Vascular Surgery, Catharina Hospital, Eindhoven, Netherlands

**Keywords:** abdominal aorta, elastography, multi-aperture, ultrasound, coherent compounding

## Abstract

**Introduction:** In this paper we introduce *in vivo* multi-aperture ultrasound imaging and elastography of the abdominal aorta. Monitoring of the geometry and growth of abdominal aortic aneurysms (AAA) is paramount for risk stratification and intervention planning. However, such an assessment is limited by the lateral lumen-wall contrast and resolution of conventional ultrasound. Here, an *in vivo* dual-aperture bistatic imaging approach is shown to improve abdominal ultrasound and strain imaging quality significantly. By scanning the aorta from different directions, a larger part of the vessel circumference can be visualized.

**Methods:** In this first-in-man volunteer study, the performance of multi-aperture ultrasound imaging and elastography of the abdominal aortic wall was assessed in 20 healthy volunteers. Dual-probe acquisition was performed in which two curved array transducers were aligned in the same imaging plane. The transducers alternately transmit and both probes receive simultaneously on each transmit event, which allows for the reconstruction of four ultrasound signals. Automatic probe localization was achieved by optimizing the coherence of the trans-probe data, using a gradient descent algorithm. Speckle-tracking was performed on the four individual bistatic signals, after which the respective axial displacements were compounded and strains were calculated.

**Results:** Using bistatic multi-aperture ultrasound imaging, the image quality of the ultrasound images, *i.e.*, the angular coverage of the wall, was improved which enables accurate estimation of local motion dynamics and strain in the abdominal aortic wall. The motion tracking error was reduced from 1.3 mm ± 0.63 mm to 0.16 mm ± 0.076 mm, which increased the circumferential elastographic signal-to-noise ratio (SNRe) by 12.3 dB ± 8.3 dB on average, revealing more accurate and homogeneous strain estimates compared to single-perspective ultrasound.

**Conclusion:** Multi-aperture ultrasound imaging and elastography is feasible *in vivo* and can provide the clinician with vital information about the anatomical and mechanical state of AAAs in the future.

## 1 Introduction

Ultrasound imaging is widely used in clinical practice to monitor the size and growth of abdominal aortic aneurysms (Wanhainen et al., 2020). When the aortic diameter exceeds 5.0 cm in women and 5.5 cm in men, or when the growth rate exceeds 1 cm/year, surgical intervention is performed (Wanhainen et al., 2020). However, this threshold is based on population statistics (Brewster et al., 2003), and previous studies have highlighted the limitation of purely morphological assessment for the estimation of the rupture risk (Darling et al., 1977; Raut et al., 2013). The knowledge of the full aneurysm geometry as well as the wall motion dynamics and strain can give valuable insights in the mechanical state of the abdominal aortic wall, and contribute to a more patient-specific rupture risk assessment (Bihari et al., 2013; Karatolios et al., 2013; Wittek et al., 2013; Van Disseldorp et al., 2016).

However, the use of conventional ultrasound for the reconstruction of aortic geometry and estimation of wall motion and strain has its limitations. Tissue interfaces perpendicular to the propagation direction of the ultrasound wave reflect well, whereas at interfaces parallel to the propagation direction the ultrasound wave may reflect away from the transducer (Petterson et al., 2021). As a result, the anterior and posterior side of the vessel wall can be well visualized by the specular reflections, but there is limited to poor contrast and resolution at the lateral sides. This is also illustrated in (Matthews et al., 2021), where it is shown that the reproducibility of aortic diameter measurements in the transverse direction is significantly lower compared to the anterior-posterior plane. To overcome these physical limitations of ultrasound imaging, the aorta can be scanned from multiple directions to increase the visibility of the entire circumference of the aortic wall by specular reflections. In Petterson et al. (2021), this concept of multi-perspective ultrasound imaging, in which an ultrasound probe was physically translated over the abdomen, improved lateral contrast of the abdominal aorta in healthy volunteers. In a later study (Sjoerdsma et al., 2023), 3D multi-perspective ultrasound imaging was also performed in patients with an abdominal aortic aneurysm. Also in obstetric (Zimmer et al., 2023) and cardiac applications (Rajpoot et al., 2011; Mulder et al., 2014), the use of multi-perspective imaging has shown to improve the visibility of the imaged structures. However, in such an approach, the ultrasound images have to be temporally aligned to match the cardiac cycle. This can be problematic because of the changes in the hemodynamics between acquisitions.

Recent developments in ultrafast imaging (Tanter and Fink, 2014) have enabled the use of multiple apertures during one acquisition, by performing interleaved scanning (Peralta et al., 2019; de Hoop et al., 2020; Liu et al., 2022a), or in a configuration that enables the transmission of one coherent wavefront using all apertures at the same time (Foiret et al., 2022). In these studies, multiple linear or curved array transducers were used, typically driven by research ultrasound platforms, to develop the multi-aperture imaging systems. Dual-aperture ultrasound also allows for bistatic imaging, a concept that originates from radar technology (Burkholder et al., 2003), in which one transducer transmits and a second transducer receives. This concept has previously also been used in for instance Doppler imaging, to obtain more reliable velocity estimates (Dunmire et al., 2000). In (Van Hal et al., 2021), dual-aperture bistatic ultrasound imaging of the abdominal aorta allowed for the reconstruction of four ultrasound signals, since both transducers received simultaneously upon each transmit event. Using this dual-receive image acquisition configuration, the visibility of the aortic wall circumference in simulations and *ex vivo* porcine aortas was increased by 200%.

Multi-aperture ultrasound imaging cannot only improve ultrasound image quality, but it can also improve the assessment of tissue deformation and strain (Van Hal et al., 2021; Liu et al., 2022b). Conventional strain imaging techniques are limited by the lack of phase information and resolution in the lateral direction of the ultrasound beam (Lopata et al., 2009b; Hansen et al., 2010). Phase information in the lateral direction can be added by the use of beam steering (Techavipoo et al., 2004; Hansen et al., 2010), however, this technique cannot be applied to the abdominal aorta or deeper lying structures in general, considering the limited overlapping region in compounding at large depths (Petterson et al., 2021). This insufficient overlap can be overcome by the use of multiple probes, as shown in (Van Hal et al., 2021; Liu et al., 2022b). The addition of the second transducer improved accuracy and precision of radial and circumferential strain estimates in aortic and cardiac applications. However, so far all these studies were conducted in *ex vivo* experiments. *In vivo* application of ultrafast multi-aperture ultrasound imaging have been shown in (Peralta et al., 2020; Foiret et al., 2022), in which its performance in terms of increased field-of-view (FOV) and improved resolution was shown in the liver of a healthy volunteer.

In this study, the advantage of using multiple apertures over conventional single-transducer imaging of the abdominal aorta is assessed *in vivo* in 22 healthy volunteers. The image quality of bistatic multi-aperture ultrasound imaging is compared to single-perspective ultrasound imaging, focusing on the visibility of the abdominal aortic wall and surrounding tissues. Moreover, bistatic multi-aperture strain imaging is performed *in vivo* for the first time to assess the improvement in the estimation of wall motion and strain. *In vivo* evaluation of the developed algorithms for image reconstruction, image registration and fusion, and strain imaging is important since physical conditions will be different and more challenging than those experienced in experimental and simulated set-ups. For instance, there are additional challenges considering the larger imaging depth and speed-of-sound differences in the abdominal wall. This also makes accurate estimation of the probe positions more difficult, which is essential for the reconstruction and fusion of multi-aperture bistatic ultrasound images. In previous research, probe localization relied on detectable and isolated point scatterers (Peralta et al., 2019), inherent image features (Petterson et al., 2021) or the reflection coming from the lens of the second transducer (De Hoop et al., 2020; Van Hal et al., 2021). We have now developed a more generic method for probe localization, based on maximizing signal coherence. Finally, our set-up allowed for flexible positioning of the probes, to obtain the best probe angle for each individual subject based on the acquired images. The influence of this relative probe positioning on the ultrasound image quality is also investigated.

## 2 Materials and methods

### 2.1 Ultrasound acquisition

**FIGURE 1:**
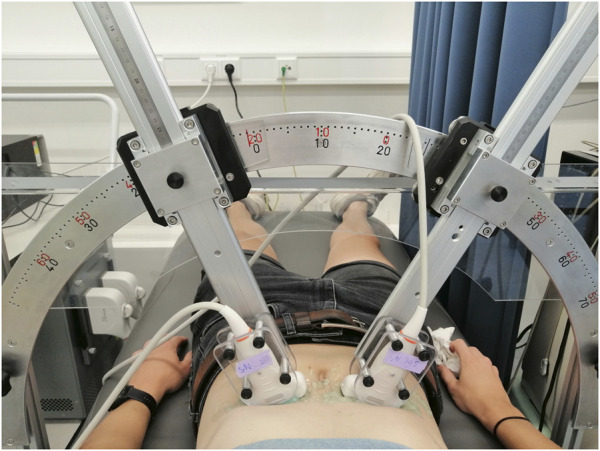
Experimental arch set-up used to perform the *in vivo* measurements of the abdominal aorta.

The feasibility and performance of bistatic multi-aperture ultrasound imaging and strain imaging of the abdominal aorta was tested in 22 healthy volunteers, between 18–60 years of age. The experiment protocol was approved by the ethical review board of the Eindhoven University of Technology and written informed consent was obtained prior to scanning. Measurements were performed using the Verasonics Vantage 256 (Kirkland, Seattle, WA, United States), equipped with two C5-2v curved array transducers with a center frequency of 3.7 MHz. A customized arch was designed on which the probe holders were attached (Figure 1). This mechanical arch allows for free positioning of the probes under a certain angle, while keeping them in the same imaging plane. After adjusting for the optimal position of the transducers, based on the images acquired, the data were recorded for approximately 3 cardiac cycles during breath-hold. The acquisition parameters for dual-aperture ultrafast imaging were based on the experiences in (Van Hal et al., 2021) and chosen according to a trade-off between the image quality and frame rate (Alomari et al., 2014), and to prevent signal decorrelation between the two transducers. An interleaved acquisition scheme was used using 15 diverging waves per transducer with a pulse repetition frequency of 4 kHz. Hence, the time between transducers was equal to 3.75 ms. Transmit apodization by a Tukey window was applied to all transducer elements to reduce the intensity of the side-lobes. The transmit angles, defined as the angle of the transmit beam with the z-axis at the location of the transducer origin, were set between −12° and 12°. The resulting frame rate for each transducer was equal to 130 Hz. The acquisition parameters are summarized in Table 1.

**TABLE 1:** Acquisition parameters.

Setting	Value
Center frequency C5-2v probe	3.7 MHz
Steering angles	[−12°, 12°]
Frame rate	130 Hz
Pulse repetition frequency	4 kHz
Number of transmits per probe	15

The mechanical safety of the ultrafast acquisition schemes was guaranteed, by measuring the mechanical index (*MI*), spatial peak pulse average intensity (*I*_*SPPA*_), and spatial peak time average intensity (*I*_*SPTA*_) using a dedicated water tank system (Precision Acoustics Ltd, Dorchester, UK). A hydrophone (Onda HNP-0400) was used to measure acoustic pressure, and could be positioned at any desired measurement location using the UMS Control Software. The measurements were performed for different transmit voltages, steering angles, pulse repetition and transmit frequencies. The results were compared against the FDA limits for abdominal ultrasound imaging after deration at a rate of 0.3 dB/cm/MHz. Moreover, the surface temperature of the curved array transducer was monitored in free air and on a test object, according to IEC 60601-2-37 (NEN-EN-IEC 60601-2-37:2008/A1:2015, 2015). All values used in this study are under the FDA regularoty limits for *MI* of 1.9, *I*_*SPPA*_ of 190 *W*/*cm* ^2^ and *I*_*SPTA*_ of 94 *mW*/*cm* ^2^. Furthermore, there were no limits for the maximum scanning time according to the surface temperature measurements. For interleaved dual-transducer imaging, the same regulatory limits apply as for conventional ultrasound imaging, since only one transducer is active at the same time.

### 2.2 Image registration and fusion

Registration and fusion of the images from the two ultrasound probes was performed in postprocessing after the acquisition of the ultrasound data. All image processing operations were performed in MATLAB 2021a (The MathWorks, Natick, MA, United States). In order to align the ultrasound images from the two probes and for the reconstruction of the signals transmitted by one transducer and received by the other transducer (the trans-probe data), accurate probe localization is essential. For this purpose, the IQ data were reconstructed on a Cartesian grid with a spacing of $\frac{1}{2}$ wavelength (*λ*) in both directions.

The probe localization method is based on maximizing the signal coherence of the compounded *T*_1_ *R*_2_ signals from all transmit angles. Since the coherence optimization therefore focuses both in transmit and receive, it can be expected that the signal power increases towards the correct probe positions. The negative signal power was used as the objective function for a gradient descent algorithm to find the optimum geometric transformation **T**, parameterized by two translational and one rotational degree of freedom. The definition of the negative signal power *P* ^−^ is given in Eq. 1, where *N* is the number of pixels in the imaged region, and *A*_*i*_ the amplitude of a pixel in the envelope detected *T*_1_ *R*_2_ image.

$$P^{-}=-\overset{N}{\underset{i=1}{\sum }}|A_{i}{|}^{2}$$ (1)

**FIGURE 2:**
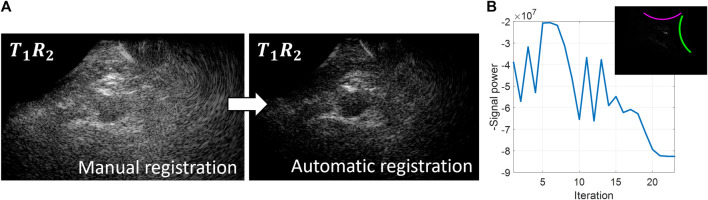
Overview of the probe localization method. A gradient descent algorithm is used to optimize the signal coherence of the *T*_1_ *R*_2_ signals from all transmit angles. **(A)** Coherently compounded *T*_1_ *R*_2_ signals from all transmit angles using manual registration of the probe locations (left) and automatic registration of the probe locations based on coherence optimization (right). **(B)** Optimization function during the gradient descent iteration process.

An overview of the probe localization method is provided in Figure 2. The numerical gradient was evaluated in each of the three degrees of freedom simultaneously, to update the estimated probe positions from the previous iteration by multiplying it with learning rate *μ* (Eq. 2).

$$T⟵T-\mu \nabla_{T}\left(P^{-}\left(T\right)\right)$$ (2)

Initialization of the gradient descent algorithm was performed using the probe angle that could be read from the customized arch. After rotating the images based on this probe angle, the images from the two probes were registered manually based on the location of the aorta to obtain the initial translation parameters. As can be seen in Figure 2, these initial registration parameters did not result in optimum image quality and signal power of the *T*_1_ *R*_2_ image. During the gradient descent optimization process, the image quality of the *T*_1_ *R*_2_ image is improved. Convergence of the gradient descent algorithm was determined by the value of the negative signal power. Convergence was achieved when the negative signal power remained constant (within a 5% range of the difference compared to the starting value) for a minimum of 3 iterations. Since initialization was performed by manual registration of the images, less than 50 iterations were needed for the gradient descent algorithm to converge to the optimum relative probe positions. Using a GPU-accelerated framework for the delay-and-sum beamforming on a NVIDIA GeForce GTX 1650^®^, the registration parameters were obtained in the order of 10–20 min.

After obtaining the relative probe positions, the individual four signals in bistatic ultrasound imaging were combined by means of coherent compounding on a grid with a spacing of $\frac{1}{4}\lambda$ , using Eq. 3.

$$I_{\mathrm{bistatic}}=I_{T_{1}R_{1}}+I_{T_{2}R_{2}}+\frac{1}{2}\left(I_{T_{1}R_{2}}+I_{T_{2}R_{1}}\right)$$ (3)

Here, *T* indicates the transmitting transducer, that is either 1 or 2 and *R* indicates the receiving transducer, that is either 1 or 2. The two trans-probe signals, which are the signals transmitted by one transducer and received by the other transducer, *i.e.*, *T*_1_ *R*_2_ and *T*_2_ *R*_1_, contain similar information because of acoustic reciprocity (Devaney, 2012), therefore their relative contribution was averaged before summation with the single-probe signals (*T*_1_ *R*_1_ and *T*_2_ *R*_2_).

### 2.3 Bistatic multi-aperture strain imaging

For the purpose of displacement estimation, the received IQ data were reconstructed on a sector grid with respect to the receiving transducer. It consisted of 2 lines per pitch in the lateral direction (pixel spacing between 0.41–0.54 mm/pixel, depending on the imaging depth), and a pixel spacing of $\frac{1}{8}\lambda$ (0.051 mm/pixel) in the axial direction. With curved array transducers, the sector grid is the preferred reconstruction grid, since it allows for the estimation of displacements along the ultrasound beam direction.

Frame-to-frame displacement estimation was performed on the individual four signals using a 2-D coarse-to-fine speckle-tracking algorithm (Lopata et al., 2009b). Similar to (Van Hal et al., 2021), the coarse displacements were estimated on the envelope data, using a kernel size of 2.6 mm × 4.5–5.9 mm (axial × lateral). Secondly, the fine displacements were estimated on the RF-data using a kernel size of 0.8 mm × 2.1–2.7 mm. The axial kernel sizes were determined with respect to the wall thickness of the aorta. The coarse axial kernel size was set to cover the full wall thickness, while the fine kernel size was set to approximately half the wall thickness to be able to measure radial strain. The lateral kernel size was set to be at least as large as the estimated lateral resolution at the highest tracking depth [around 2.5 mm (Van Hal et al., 2023)], to include enough characteristic image features for motion tracking (De Hoop et al., 2020). These are much larger than the axial kernel sizes because of the limited resolution in this direction. The search area was defined to be 3.0 mm × 4.9–6.5 mm for the coarse displacements and 1.0 mm × 2.5–3.2 mm for the fine displacements. Sub-resolution displacement estimations were improved by parabolic interpolation of the cross-correlation function (Céspedes et al., 1995; Lopata et al., 2009b).

As a last step, the axial and lateral displacements were filtered with a median filter of 0.6 mm × 4.5–5.9 mm (11 × 11 pixels).

**FIGURE 3:**
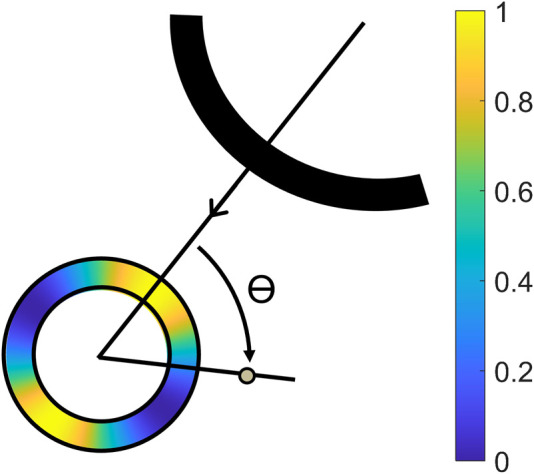
Illustration of the projection angle *θ* between the axial direction of the receiving transducer, and the radial direction of the aorta, with a color overlay of the mask created by Eq. 4 (Van Hal et al., 2021).

The axial displacement fields *u*_*ax*_ were radially projected according to *u*_*rad*_ = *u*_*ax*_/cos *θ*, in which *θ* represents the angle between the axial direction of the ultrasound transducer and the radial direction of the aorta (Hansen et al., 2010). The radial displacements were then compounded using the angular compounding technique presented in (Hansen et al., 2010; De Hoop et al., 2020; Van Hal et al., 2021). The lateral displacements were not used because of the poor lateral resolution, and the lack of phase information in this direction. Eq. 5 describes the formula that was used to weigh the radial displacement fields *u*_*rad*_ from each of the four signals in bistatic imaging. Angular weighted normalized masks $\hat{M}$ were defined to retrieve the most accurate displacement estimations from each ultrasound signal. The masks were created using a cosine function (De Hoop et al., 2020; Van Hal et al., 2021), and the result is illustrated in Figure 3:

$$M_{i,j}=\frac{1}{2}\cos\left(2\theta_{i,j}\right)+\frac{1}{2}.$$ (4)

Cut-out regions between the 70° and 110° angle were used to prevent infinite radial displacements. The masks were aligned with the axial direction of the receiving transducer in *T*_1_ *R*_1_ and *T*_2_ *R*_2_, and the wall segments between the transducers in *T*_1_ *R*_2_ and *T*_2_ *R*_1_. Again, the radial displacements fields from *T*_1_ *R*_2_ and *T*_2_ *R*_1_ were averaged, because these signals contain similar image information.

$$\begin{array}{ll} u_{\mathrm{rad}} & ={\hat{M}}_{T_{1}R_{1}}\left(u_{rad,T_{1}R_{1}}\right)+{\hat{M}}_{T_{2}R_{2}}\left(u_{rad,T_{2}R_{2}}\right)+\frac{1}{2}{\hat{M}}_{T_{1}R_{2}}\left(u_{rad,T_{1}R_{2}}\right)\\ & \;+\frac{1}{2}{\hat{M}}_{T_{2}R_{1}}\left(u_{rad,T_{2}R_{1}}\right) \end{array}$$ (5)

The resulting compounded displacement field was applied to a segmentation of the vessel wall to track the wall motion over all frames. The vessel wall segmentation was created by manual segmentation of the lumen-wall border on the bistatic ultrasound image. The corresponding points on the outer border were found by extrapolating the selected points with a uniform wall thickness of 1.7 mm, based on estimations from previous research (Slobodin et al., 2016).

Finally, for the estimation of the strains, the displacements of the tracked vessel wall coordinates were calculated with respect to the first frame, after which a 2D least-squares strain estimator, described in Lopata *et al.* (Lopata et al., 2009a) was applied using a strain kernel of 5 radial × 5 circumferential mesh points (1.7 mm × 2.6 mm).

### 2.4 Data analysis

The generalized contrast-to-noise ratio (gCNR) (Rodriguez-Molares et al., 2019) was computed as a robust measure for the contrast between the lumen and the vessel wall. A binary mask of the vessel wall segmentation was used as ROI for the vessel wall. The lumen ROI was also created using the coordinates of the lumen-wall segmentation. However, the binary mask was eroded by a disk-shaped structuring element with a radius of 0.6 mm to ensure that the specular reflections from the vessel wall were not included. For the calculation of the gCNR, the envelope detected image data were used.

The ROI of the vessel wall was divided in 8 sections of 45°, to allow for a regional analysis of the gCNR across the vessel’s circumference. Region 1 and region 5 were defined to be aligned with the beam direction of the right transducer. The signals transmitted and received by this transducer were also used as single-perspective configuration for comparison. The other regions were defined in a clockwise manner.

For the analysis of the tracking performance, the mean drift error (ME) was used. This error was calculated as the distance between the *n* points in the middle wall layer of the vessel segmentation at the start and the end of a complete cardiac cycle, at end-diastole. With a higher precision of the estimated displacements, the ME decreases, since the aortic wall comes back to the same position after tracking one cardiac cycle. The ME is defined in Eq. 6, where *x*_*i*_ and *z*_*i*_ denote the positions in the aortic wall at the starting frame, *i.e.*, first end-diastole or begin systole (bs) and ending frame, *i.e.*, second end-diastole (ed). These two frames were selected from the M-mode of the acquired ultrasound frames.

$$\text{ME}=\frac{1}{n}\overset{n}{\underset{i=1}{\sum }}\sqrt{{\left(x_{i,bs}-x_{i,ed}\right)}^{2}+{\left(z_{i,bs}-z_{i,ed}\right)}^{2}}$$ (6)

The strain estimation precision was quantified by the elastographic signal-to-noise ratio (SNRe), defined by Eq. 7, using the mean (*μ*_*ɛ*_) and the standard deviation (*σ*_*ɛ*_) of the radial and circumferential strain estimates as measured in the middle layer of the segmented wall. This value is expected to be larger in the regional analysis of the results, compared to the global results, since a natural variation is also present in a completely correct estimation due to the spine’s influence, which will lower the SNRe.

$$SNR_{e}=20\log_{10}\frac{\mu_{\varepsilon }}{\sigma_{\varepsilon }}$$ (7)

In the evaluation of the strain results, a small section (ranging between 35° and 40° wide) of the vessel wall segmentation at the left and right wall were left out, because in these regions the bistatic technique still does not add sufficient signal and contrast is low.

To compare the means of these metrics between single-perspective and bistatic multi-aperture imaging across the 8 regions in the vessel wall, paired t-tests performed. If the differences were not normally distributed, a paired Wilcoxon signed rank test was used. Differences were considered significant for *p* < 0.05.

## 3 Results

**FIGURE 4:**
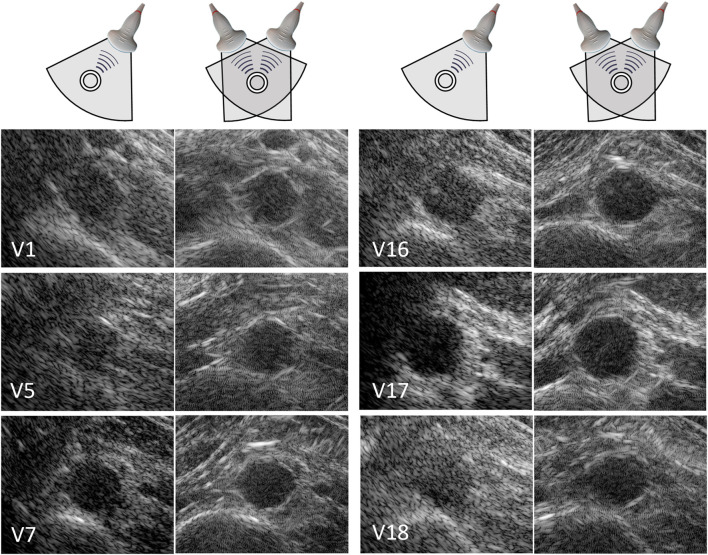
Single-perspective (left), and coherent dual-aperture bistatic (right) images of the abdominal aorta in 6 volunteers (V1, V5, V7, V16, V17, and V18). All ultrasound images are displayed using a 50 dB dynamic range. The size of the zoomed windows are 5 cm in the x-direction and 4 cm in the z-direction.

**FIGURE 5:**
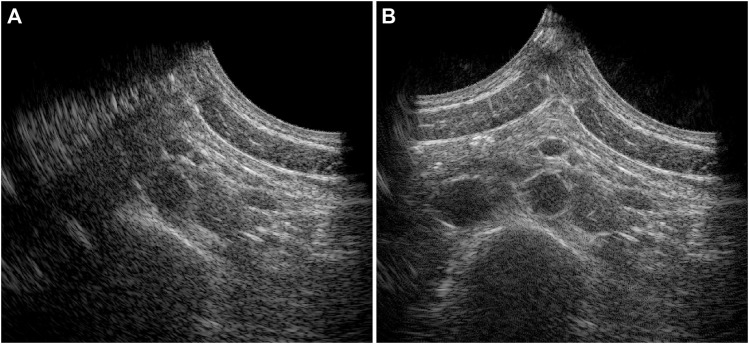
Full field-of-view images of the single-perspective **(A)** and bistatic dual-aperture ultrasound acquisitions **(B)** of the abdominal aorta in volunteer 1.

In 20 out of the 22 volunteers, the acquisition was performed without problems. The datasets of 2 volunteers were excluded, because of a poor imaging window. The single-perspective and bistatic coherent dual-aperture ultrasound images of the abdominal aorta in 6 volunteers are visualized in Figure 4. It can be seen that using dual-aperture ultrasound imaging, a larger part of the vessel wall is visible. Moreover, in Figure 5, it is shown that the second transducer also enables a larger field-of-view. In Figure 5A, the vena cava was not visible because of acoustic shadowing. But in Figure 5B, the vena cava, the shape of the vertebra, and the abdominal muscles show improved visibility.

**FIGURE 6:**
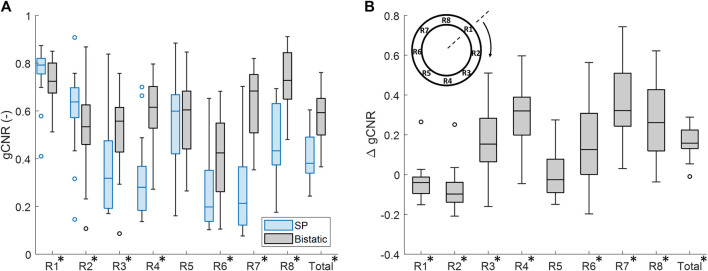
Regional analysis of the generalized contrast-to-noise ratio (gCNR) between the vessel wall and the lumen of the aorta in all volunteers. **(A)** gCNR in single-perspective (SP) and multi-aperture bistatic ultrasound imaging in the 8 regions of the vessel wall. **(B)** Difference in gCNR (ΔgCNR) for bistatic imaging compared to single-perspective ultrasound imaging. Regions in which the differences were statistically significant have been indicated with an asterisk in the label.

The vessel-lumen gCNR over all volunteers is quantified in Figure 6, which shows a significant increase of 0.16 ± 0.15 (40%) when using dual-aperture bistatic ultrasound imaging compared to single-perspective ultrasound, when considering the entire circumference of the aortic wall (*p* < 10^–8^). When analyzing the 8 regions individually, it can be seen that the most information is added at the lateral sides of the single-perspective ultrasound configuration (R3 and R7) and at the vessel wall regions between the transducers (R4 and R8). In the latter regions, the reflections on the vessel wall originate from the trans-probe image data. In R3, R4, R7, and R8, the mean increase in gCNR was equal to 0.27 ± 0.23 (78%). However, in R1 and R2, there was a small decrease in gCNR of −0.059 ± 0.20 (−8.6%) using bistatic imaging compared to single-perspective ultrasound. These regions correspond to the anterior part of the vessel wall that was also well visible in the single-perspective signal configuration. In the corresponding posterior part of the vessel wall (R5), the mean gCNR remained almost the same, comparing multi-aperture bistatic and single-perspective ultrasound, with a value of 0.57 ± 0.15. The differences between single-perspective and multi-aperture bistatic imaging were significant in all regions except R5 (*p* < 0.01), which is indicated in Figure 6.

Similar results were found if the left probe was used for single-perspective imaging as a small decrease in gCNR of −0.096 ± 0.24 (−16%) was observed symmetrically in R6 and R7. However, the overall gCNR across the circumference of the aorta was also increased by 0.18 ± 0.15 (47%).

**FIGURE 7:**
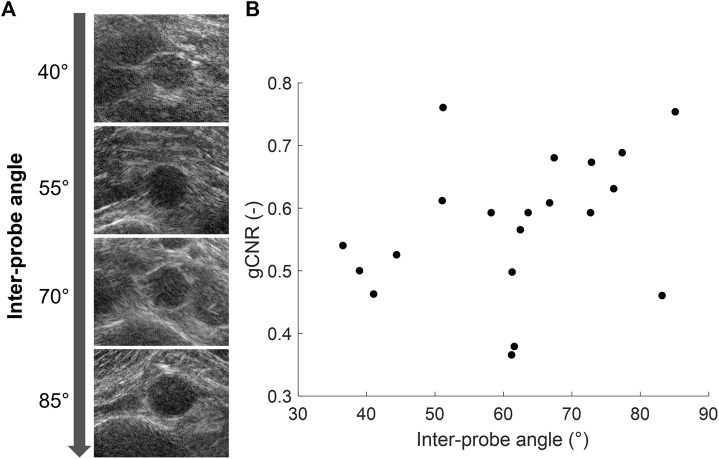
**(A)** Coherent dual-aperture bistatic ultrasound images of the abdominal aorta from different volunteers obtained with increasing inter-probe angles. **(B)** Generalized vessel-lumen contrast-to-noise ratio (gCNR) across the entire circumference of the aorta in each of the 20 volunteers, plotted against the used inter-probe angle during the acquisition.

In each volunteer, the ultrasound images were acquired with different probe positions based on the best available imaging window. To investigate the influence of this relative probe positioning on the ultrasound image quality, the measured vessel-lumen gCNR in each volunteer was plotted against the used inter-probe angle during the acquisition in Figure 7. Here, it is shown that a larger vessel-lumen gCNR can be obtained when the ultrasound probes are positioned under an angle closer to 90°, since it results in a larger part of the vessel circumference to be visible by specular reflections. Next to the inter-probe angle, the gCNR also seemed to be dependent on the imaging depth, as higher gCNR values were obtained at smaller imaging depths. The two datasets with a large imaging depth (9 cm and 13 cm, respectively), and an inter-probe angle of around 60°, led to lower gCNRs of 0.37 and 0.38. However, in one dataset an inter-probe angle close to 85° also resulted in a relatively low gCNR of 0.46, despite the small imaging depth of 4.7 cm.

**FIGURE 8:**
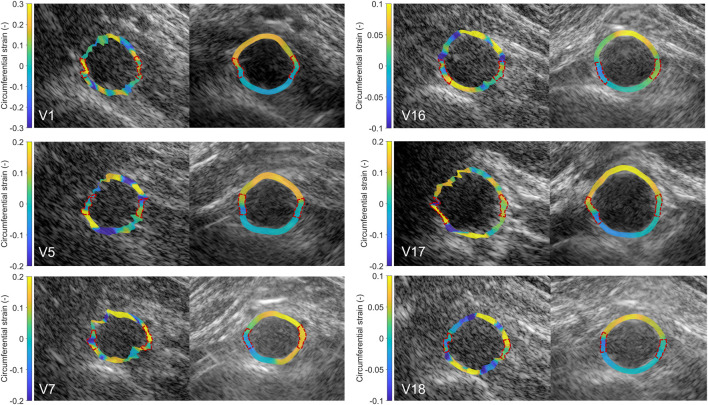
Estimated local circumferential strain in the aortic vessel wall at systole using single-perspective (left), and dual-aperture bistatic (right) ultrasound imaging in 6 volunteers (V1, V5, V7, V16, V17, and V18). The indicated sections at the right and left side of the wall were left out of the analysis. The size of the zoomed windows are 5 cm in the x-direction and 4 cm in the z-direction. A video corresponding to this figure is available as Supplementary Material.

After performing fusion of the individual bistatic signals, functional imaging was performed. In Figure 8, the circumferential strain results at end-systole are shown for the same volunteers as in Figure 4. The estimation of local strains in the vessel wall using single-perspective ultrasound leads to a noisy pattern. However, by compounding the axial displacements from different directions, more homogeneous local strains are obtained, showing a high level of continuity without the use of regularization. Over all volunteers, a clear strain pattern is found with lower circumferential strains at the posterior side, and higher circumferential strains at the anterior side. In some volunteers, like volunteer 7 (V7) and V16, the circumferential strains were also higher on the right side, compared to the left side because of the location of the spine.

**FIGURE 9:**
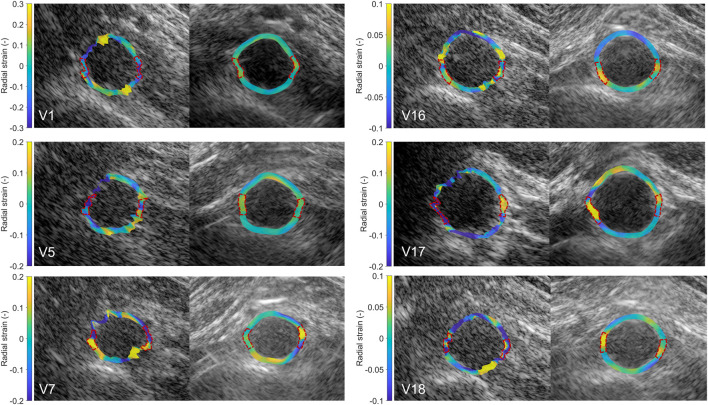
Estimated local radial strain in the aortic vessel wall at systole using single-perspective (left), and dual-aperture bistatic (right) ultrasound imaging in 6 volunteers (V1, V5, V7, V16, V17, and V18). The indicated sections at the right and left side of the wall were left out of the analysis. The size of the zoomed windows are 5 cm in the x-direction and 4 cm in the z-direction. A video corresponding to this figure is available as Supplementary Material.

In Figure 9, the radial strain pattern is shown corresponding to the examples in Figure 8. It is shown that the radial strain pattern becomes more homogeneous compared to the single-perspective imaging results. In some volunteers, like in V1 and V5, almost no radial strain was measured. However, in other volunteers, e.g., V7 and V16, lower radial strains were found at the anterior side of the vessel wall, and higher radial strains were found at the posterior side of the vessel wall.

**FIGURE 10:**
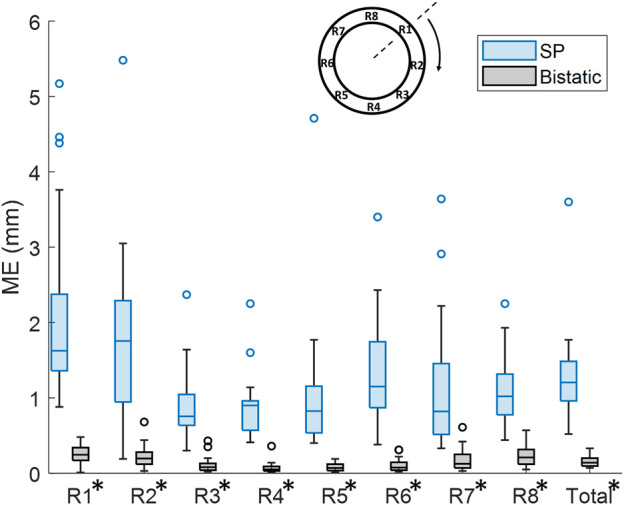
Regional analysis of the tracking error (ME) between single-perspective and bistatic imaging configurations in all volunteers. All differences were statistically significant (p < 10^–4^), which is indicated with an asterisk in the label.

By using multiple apertures, the overall mean tracking error was significantly reduced from 1.3 mm ± 0.63 mm to 0.16 mm ± 0.076 mm, which is shown in Figure 10 (*p* < 10^–4^). In the regional analysis of the tracking error, it can be seen that the tracking error using bistatic ultrasound imaging was slightly larger in the anterior regions, compared to the posterior regions of the vessel wall. This is also related to the difference in expansion between the anterior and posterior side, as expansion of the vessel wall at the posterior side is limited by the spine.

**FIGURE 11:**
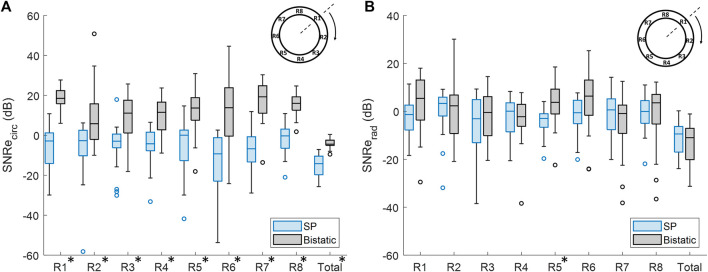
Regional analysis of the elastographic signal-to-noise ratio (SNRe) between single-perspective and bistatic imaging configurations in all volunteers. Regions in which the differences were statistically significant have been indicated with an asterisk in the label. **(A)** Circumferential SNRe **(B)** Radial SNRe.

In Figure 11A, it is shown that the overall mean SNRe^circ^ was significantly increased by 12.3 dB ± 8.3 dB, using bistatic ultrasound imaging (*p* < 10^–5^). Also when looking at each of the 8 regions individually, a significant increase in SNRe^circ^ was observed using multi-aperture bistatic imaging compared to single-perspective imaging (*p* < 0.01).

The change in SNRe^rad^ using bistatic ultrasound imaging compared to single-perspective ultrasound was not significant, except for R5, as shown in Figure 11B. In Figure 9, it can be seen that a certain amount variation inside the vessel wall remained after displacement compounding. The largest increase in mean SNRe^rad^ was found in R1 and R5, by 5.3 dB ± 16.9 dB and 6.6 dB ± 11.8 dB, respectively.

## 4 Discussion

In this study, the feasibility and performance of ultrafast dual-aperture bistatic ultrasound imaging and elastography of the abdominal aorta was tested for the first time *in vivo* on 20 healthy volunteers. It was shown that the 4 signals obtained with 2 transducers can be coherently compounded despite *in vivo* challenges, to create a better image of the abdominal aorta and its surroundings, *i.e.*, visualizing a larger part of the vessel wall with higher contrast. The addition of multidirectional high-resolution phase information also allows for improved local strain estimation because displacements can be estimated accurately in more directions.

The registration of the individual datasets was performed with the help of a customized arch, that allowed for read-out of the used probe angle. Optimization of the initial registration parameters could be performed fully automatically, without the use of inherent image features. Currently, an appropriate initialization was still necessary because multiple local minima could be found in the signal power of the trans-probe data. We also found out that in some cases, the global minimum did not correspond to the best alignment of the aorta, which is likely due to aberrations and artifacts involved. To improve the image quality by coherent compounding even more, aberration correction can be performed using for instance the methods presented in (Van Hal et al., 2023) or (Foiret et al., 2022). In the future, it might be possible to perform aberration correction and registration at the same time, since the correction for differences in speed-of-sound also leads to better alignment of the imaged structures. This will also improve the resolution in the final bistatic ultrasound image even more, compared to the results shown in Figures 4, 5, since improved coherence of the signals from multiple probes will lead to smaller point spread functions (Foiret et al., 2022; Van Hal et al., 2023). Future research could also focus on the optimization procedure to further automate this process, and to make it faster, since each gradient descent iteration required six reconstructions: two for each degree of freedom to be optimized.

Even without the use of aberration correction, this study has shown that the image quality and strain results were significantly improved compared to single-perspective ultrasound. The gCNR between the vessel wall and the lumen was found to increase by 40% on average, using bistatic ultrasound imaging compared to single-perspective ultrasound. Largest improvements were found at the lateral sides of the single-transducer configuration, i.e., R3 and R7, and also the vessel wall regions between the transducers, i.e., R4 and R8. However, in R1 and R2, which correspond to the anterior part of the vessel in the axial direction of the single-transducer configuration, there was a small decrease in vessel-lumen gCNR. This can be easily explained since these regions were already well visible in the single-perspective configuration. Hence, the added signals from the second transducer contributed less image information since R1, R2, and R5 correspond to its lateral direction where there is less contrast. In the future, smart compounding strategies can be investigated to further improve the coherent compounded result. In (Van Hal et al., 2021), different weighted compounding strategies were tested that successfully optimized the vessel-lumen contrast. We also tested these strategies on the *in vivo* data, however, the used masks were not tailored to optimize the contrast in the surrounding tissues. Therefore, simple coherent compounding by Eq. 3 was preferred to optimize the ultrasound image quality as a whole, at the cost of a slight decrease in gCNR in R1 and R2.

The resulting contrast of the abdominal aorta is dependent on both the imaging depth and the inter-probe angle. In general, an angle close to 90° between the transducers is desired since it results in a larger coverage of the vessel wall by specular reflections, as measured by the vessel-lumen gCNR, shown in Figure 7. Of course in clinical practice, the optimal probe angle is mostly determined by the viewing window that depends on each patient’s anatomy and for instance the presence of bowel gas. There was one case in which the gCNR was relatively low with a large probe angle and small imaging depth, which was not expected. In this dataset, the trans-probe signals appeared as blurred, and also affected the strain estimation in the vessel wall regions between the transducers. This implies that for widespread clinical use, a multi-aperture array with sufficient degrees-of-freedom combined with shape sensing may be required to ensure high inclusion rates and the most optimal improvements in image quality. Finally, bistatic multi-aperture ultrasound images may also benefit from some post-processing as commonly performed on most modern clinical ultrasound systems, to ensure that the image quality meets the clinician’s preferences. The results in shown in Figures 4, 5 present “raw” ultrasound images, meaning that no additional filtering is applied. This is done to ensure a fair quantitative comparison of the employed image quality metrics.

The *in vivo* strain imaging results showed that local strain estimation using single-perspective ultrasound is extremely difficult, because of the large imaging depth combined with the small wall thickness, in line with our previous *ex vivo* findings (Van Hal et al., 2021; De Hoop et al., 2020). The displacements can only be accurately determined in the axial direction of the ultrasound probe, while in the lateral direction motion drift is present. By using a second aperture, the displacements can be estimated accurately in more directions and by applying angular weighted displacement compounding, a homogeneous strain pattern can be obtained which resulted in an increase of overall circumferential SNRe^circ^ by 12.3 dB ± 8.3 dB. In the left and the right wall, small sections between 35° and 40° wide were left out of the analysis in both single-perspective and multi-aperture bistatic imaging, since these regions could still not be well visualized using the current set-up. As a result, less accurate strain estimates were obtained in these regions using bistatic imaging, similar to the single probe estimations. In the future, this can be resolved with an even larger aperture.

In this study, a coarse-to-fine, block-matching based speckle tracking algorithm was used to obtain the 2D displacement fields. Future implementation of multi-aperture bistatic imaging could potentially benefit from more advanced tracking algorithms, like Jiang and Hall (2011), which could improve the strain estimation results even more. This was out of the scope of the current study to isolate the comparison of single and multi-aperture imaging from other effects as much as possible. Moreover, this study has shown that using bistatic imaging, strain estimation can be improved by nature, without the use of any regularization.

In R1, R2, and R5 large improvements in motion tracking and SNRe^circ^ were obtained despite the similar or slightly decreased gCNR. In these regions, the circumferential direction is aligned with the lateral direction in the single-perspective imaging configuration. In the single-perspective results, no radial displacement projection was performed, hence, the large tracking errors and low SNRe^circ^ can be explained by lateral motion drift. This lateral motion drift was even worse compared to for instance R3, since R3 contained more image information in the lateral direction. In dual-aperture imaging, displacement compounding could be performed using only the estimated axial displacements from different angles, leaving out the erroneous lateral displacements. Therefore, the lateral motion drift present in the single-perspective configuration could be mitigated, which exhibited improved displacement estimation across the entire circumference of the aorta.

The resulting circumferential strain pattern using bistatic ultrasound imaging showed a clear distinction in magnitude between the anterior and posterior side of the vessel wall, that was not visible using single-perspective ultrasound imaging. This can be explained by the fact that the expansion of the aorta at the posterior side is limited by the presence of the spine and branching lumbar arteries (Goergen et al., 2007). However, in some volunteers, the circumferential strain at the posterior side was negative, which was not expected, although it is in accordance with findings from other literature (Karatolios et al., 2013; Taniguchi et al., 2014; Bracco et al., 2023). Comparing the bistatic strain patterns of V7, V16, and V18 against the single-perspective results, it may seem like the single-perspective strains in the posterior wall are more reasonable (positive circumferential strains, and negative radial strains). However, the lateral motion drift present in the single-perspective imaging configuration (as characterized by the mean error) causes overestimation (V7, V16, and V18) and underestimation (V5) as mesh nodes move toward or away of each other as a result of the accumulation of errors in the displacement estimates. The single-perspective strain results are therefore less accurate compared to the bistatic strain results. Negative circumferential strains accompanied by radial expansion, like estimated in V7, may be physiological. Otherwise, these results could be explained by incorrect assumptions in the displacement model, and out-of-plane motion. It is important to note that the estimation of local displacements was performed with respect to a fixed reference frame and midpoint. In future research, it could be more accurate to take the change in local radial and circumferential directions during the cardiac cycle into account.

The estimation of radial strain within the vessel wall still proved to be challenging. The radial strain pattern was often not homogeneous within the wall, which still resulted in similar SNRe^rad^ compared to the single-perspective results. However, especially in V16, some correspondence with the circumferential strain results was found, as visualized in Figures 8, 9. Here, it can be seen that more negative radial strains correspond with positive circumferential strains at the anterior side of the vessel wall, and lower strains are found at the posterior side of the vessel wall. The radial strains are likely less accurate compared to the circumferential strains because of the relatively low center frequency of the transducer (3.7 MHz), which limits the axial resolution (which even worsens at lower depths), combined with the small wall thickness (1.7 mm). Therefore, the difference in movement between the inner and outer wall is harder to distinguish. Moreover, the expected thinning of the aortic wall is in the same order of magnitude as the measured tracking error, which limits the precision of the radial strain estimates.

The *in vivo* strain results were limited by the lack of a ground truth. In previous research (Van Hal et al., 2021), bistatic multi-aperture strain imaging results showed good agreement with the ground truth strain obtained from ultrasound simulations. In the future, more realistic ultrasound simulations based on biomechanical models could help to fully validate *in vivo* strain patterns, incorporating *in vivo* wall geometries and properties to mimic the inhomogeneous deformation (Van Disseldorp et al., 2019; Bracco et al., 2023).

The current study was performed in healthy volunteers to show the feasibility and performance of multi-aperture bistatic ultrasound imaging and elastography *in vivo*. As a next step, the methods can be validated in AAA patients gradually moving towards the final application. In the future, we also envision a more flexible set-up, rather than the currently used experimental arch, for instance using probe holders with multiple apertures attached Zimmer et al. (2023). This will still allow for the clinician to perform a free-hand scan, and use 3-D ultrasound. These developments could in the future also contribute towards semi-tomographic ultrasound approaches that can cover a large FOV, for instance using wearable ultrasound patches (Lin et al., 2023). 3-D bistatic ultrasound imaging using two sparse matrix arrays has recently been proven feasible in an *ex vivo* study (De Hoop et al., 2022). This removes the limitation of scanning in the same imaging plane and can capture out-of-plane motion.

## 5 Conclusion

Multi-aperture ultrasound imaging and elastography and it’s feasibility *in vivo* was shown. This novel ultrasound modality can provide the clinician with much needed information about the geometry and mechanical state of the aortic wall. Using bistatic multi-aperture ultrasound imaging, both the contrast and resolution of the ultrasound images can be improved which enables accurate estimation of local motion dynamics and strain in the abdominal aortic wall. Future *in vivo* studies will focus on the practical application of multi-aperture acquisition in the clinic, in patients with AAAs, and extending our approach to full 3-D ultrasound which will allow for more free-hand operation.

## Data Availability

The datasets presented in this article are not readily available due to ethical restrictions. Requests to access the datasets should be directed to v.h.j.v.hal@tue.nl or r.lopata@tue.nl.
