# Supplementary figures and images for: In vivo bistatic dual-aperture ultrasound imaging and elastography of the abdominal aorta
Source: Front Physiol. 2024 Mar 28;15:1320456. doi: 10.3389/fphys.2024.1320456 (PMC11007781; doi:10.3389/fphys.2024.1320456)

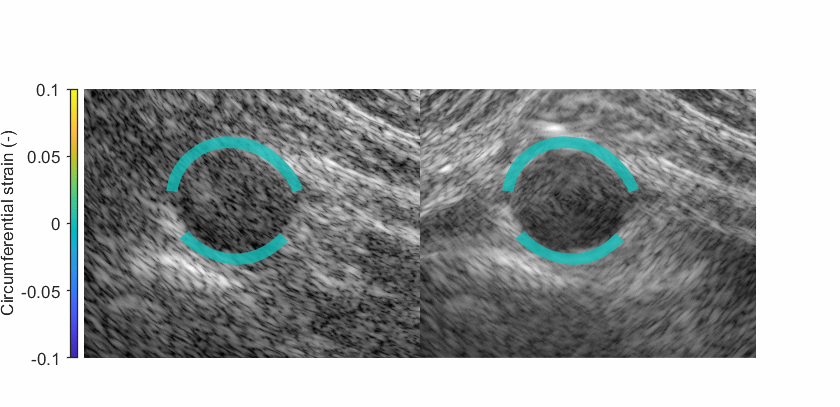

Supplement: Supplementary file 1 [file DataSheet1.zip › SupplementaryVideos/volunteer16_SPandBS_ecirc.gif]

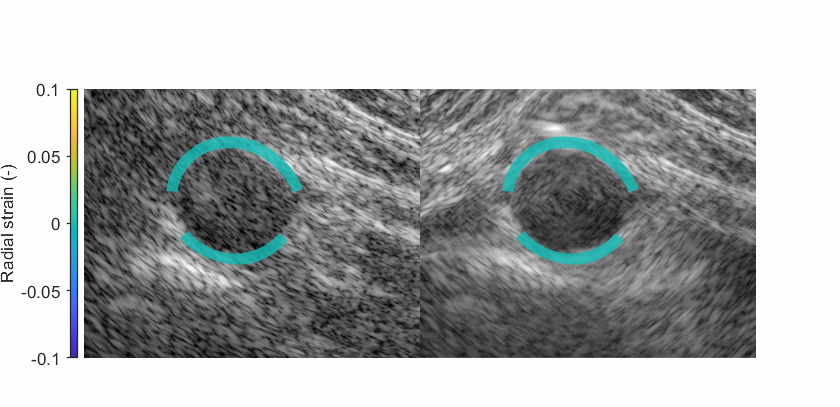

Supplement: Supplementary file 1 [file DataSheet1.zip › SupplementaryVideos/volunteer16_SPandBS_erad.gif]
